# Clinical cascades as a novel way to assess physical readiness of facilities for the care of small and sick neonates in Kenya and Uganda

**DOI:** 10.1371/journal.pone.0207156

**Published:** 2018-11-21

**Authors:** Melissa C. Morgan, Hilary Spindler, Harriet Nambuya, Grace M. Nalwa, Gertrude Namazzi, Peter Waiswa, Phelgona Otieno, John Cranmer, Dilys M. Walker

**Affiliations:** 1 Department of Pediatrics, University of California San Francisco, San Francisco, California, United States of America; 2 Institute of Global Health Sciences, University of California San Francisco, San Francisco, California, United States of America; 3 Maternal, Adolescent, Reproductive, and Child Health Centre, London School of Hygiene and Tropical Medicine, London, United Kingdom; 4 Department of Pediatrics, Jinja Regional Referral Hospital, Jinja, Uganda; 5 Department of Pediatrics and Child Health, Maseno University, Maseno, Kenya; 6 Maternal, Newborn and Child Health Centre of Excellence, School of Public Health, College of Health Sciences, Makerere University, Kampala, Uganda; 7 Department of Public Health Sciences, Karolinska Institutet, Stockholm, Sweden; 8 Center for Clinical Research, Kenya Medical Research Institute, Nairobi, Kenya; 9 School of Nursing, Emory University, Atlanta, Georgia, United States of America; 10 Department of Obstetrics, Gynecology, and Reproductive Sciences, University of California San Francisco, San Francisco, California, United States of America; Johns Hopkins School of Public Health, UNITED STATES

## Abstract

**Background:**

Globally, there were 2.7 million neonatal deaths in 2015. Significant mortality reduction could be achieved by improving care in low- and middle-income countries (LMIC), where the majority of deaths occur. Determining the physical readiness of facilities to identify and manage complications is an essential component of strategies to reduce neonatal mortality.

**Methods:**

We developed clinical cascades for 6 common neonatal conditions then utilized these to assess 23 health facilities in Kenya and Uganda at 2 time-points in 2016 and 2017. We calculated changes in resource availability over time by facility using McNemar’s test. We estimated mean readiness and loss of readiness for the 6 conditions and 3 stages of care (identification, treatment, monitoring-modifying treatment). We estimated overall mean readiness and readiness loss across all conditions and stages. Finally, we compared readiness of facilities with a newborn special care unit (NSCU) to those without using the two-sample test of proportions.

**Results:**

The cascade model estimated mean readiness of 26.3–26.6% across the 3 stages for all conditions. Mean readiness ranged from 11.6% (respiratory distress-apnea) to 47.8% (essential newborn care) across both time-points. The model estimated overall mean readiness loss of 30.4–31.9%. There was mild to moderate variability in the timing of readiness loss, with the majority occurring in the identification stage. Overall mean readiness was higher among facilities with a NSCU (36.8%) compared to those without (20.0%).

**Conclusion:**

The cascade model provides a novel approach to quantitatively assess physical readiness for neonatal care. Among 23 facilities in Kenya and Uganda, we identified a consistent pattern of 30–32% readiness loss across cascades and stages. This aggregate measure could be used to monitor and compare readiness at the facility-, health system-, or national-level. Estimates of readiness and loss of readiness may help guide strategies to improve care, prioritize resources, and promote neonatal survival in LMICs.

## Introduction

Globally, there were 2.7 million neonatal deaths in 2015 [1]. The leading causes were preterm birth, defined as birth before 37 completed weeks of gestation (16%), intrapartum-related events (11%), and sepsis or meningitis (7%) [1]. Deaths in the neonatal period are responsible for 45% of all deaths in children under age 5, with preterm birth being the leading cause [1]. Major mortality reduction could be achieved by improving care in low- and middle-income countries (LMIC) [2–5]. Within the neonatal period, 36% of deaths occur on the day of birth and 73% occur in early neonatal period, defined as the first 7 days [2]. Between 1980 and 2015, early neonatal mortality has decreased more slowly than all other age-categories of under-5 mortality [6]. Thus, the immediate postnatal and early neonatal periods represent critical windows of opportunity to improve neonatal survival globally.

Facility-based care of small and sick neonates, including neonatal resuscitation, kangaroo mother care (KMC), intravenous (IV) fluids, feeding support, oxygen, antibiotics, and phototherapy, could avert an estimated 580,000 neonatal deaths annually [7]. Estimates suggest that provision of available interventions in facilities could decrease prematurity, intrapartum, and infection-related causes of neonatal mortality by 58%, 79%, and 84%, respectively [7]. Conversely, some of these interventions also carry a risk of harm when administered by inadequately trained staff or without proper equipment. For example, provision of oxygen therapy in the absence of pulse oximetry monitoring increases the risk of retinopathy and subsequent visual impairment in preterm neonates [8,9]. Although an estimated 72% of deliveries globally occurred in facilities from 2012 to 2017 [10], lack of access to delivery and postnatal care remains a challenge in LMICs [11–13]. Further, essential interventions are not successfully implemented in many LMICs due to an array of underlying constraints, including shortages of skilled providers, inadequate funding, poor distribution of newborn care services, and weak referral systems [14]. To better understand such barriers and ultimately address the functionality of a health system as a whole, facility-level capacity limitations must first be identified.

However, analyzing facility capacity has been an ongoing focus of public health for decades and competing theories exist on how to best approach such an analysis. To develop and further standardize an approach, the United Nations Children’s Fund (UNICEF) and the World Health Organization (WHO) published guidelines describing “signal functions” related to facility readiness for provision of Essential Obstetric Care (EOC) [15]. These signal functions represent a selection of key interventions used to treat obstetric complications, which classify and monitor the level of care (basic or comprehensive) being provided by a facility, rather than listing all EOC services that should be provided [15]. In 2009, EOC was replaced by Emergency Obstetric and Newborn Care (EmONC), and the list of signal functions was expanded to include neonatal resuscitation (with a bag and mask) at facilities providing basic or comprehensive levels of care [16]. Availability, use, and quality of EmONC have been suggested as pragmatic indicators to monitor and evaluate the progress of health systems towards reducing maternal and neonatal mortality [16–21], yet there have been few attempts to develop additional signal functions for neonates. One study evaluated indicators for the quality of pediatric hospital care in LMICs and found broad support among experts for several newborn indictors, including availability of tetracycline, vitamin K, parenteral antibiotics, and drugs for the prevention of mother-to-child transmission (PMTCT) of HIV [22]. In 2012, new signal functions were proposed for routine and emergency newborn care, including KMC, IV fluids, cup feeding, oxygen, antibiotics, and PMTCT [18]; however, these have not been widely adopted globally. A recent study delineated over 600 structural characteristics, including infrastructure, equipment, drugs, providers and guidelines, for facility readiness to deliver care for small and sick newborns, and work is currently underway to finalise recommendations for newborn signal functions [23].

Notably, the signal function approach is subject to limitations and recommendations for alternative approaches have led researchers to the rethink the analysis of capacity. Potter and Brough emphasized that the key components of a functioning health system (i.e., facility/staff, skills, tools) are hierarchical in nature, and depend greatly upon the availability and functionality of each other [24,25]. Applied to a clinical context, a medical intervention can only be effectively administered if the necessary infrastructure, staff, and tools are first in place. Through precise identification of resource shortages, capacity needs can be tied to specific gaps found within each step of the hierarchy [24]. The HIV treatment cascade was developed as a tool to identify gaps in care delivery and to prioritize resources with the goal of improving public health [26–28]. At each step of the cascade (e.g., diagnosis, linkage to care), patients may be lost to follow-up and, as a result, fail to access or benefit from available health interventions. The cascade approach has subsequently been applied to other areas of public health, including PMTCT [29], hepatitis C [30], diabetes [31], hypertension [32] and, most recently, emergency obstetric care [33]. The latter introduced the clinical cascade model, which highlights the fact that multiple resources are required sequentially or simultaneously in order to provide real-time patient care [33]. For example, a provider can effectively treat a sick neonate requiring immediate care only when all resources needed to identify and treat the underlying condition are simultaneously present in the facility. In the obstetric cascade study, Cranmer and colleagues assessed 44 primary care facilities in Kakamega County, Kenya and found that 39–100% had the resources required for identification, 7–57% had resources for treatment, and 0–2% had resources to monitor or modify treatment across five common maternal emergencies [33].

Informed by Potter and Brough’s capacity pyramid and based on the obstetric emergency cascade [24,33], we aimed i) to develop clinical cascades to evaluate facility readiness to care for small, sick neonates and ii) to utilize these to assess 23 health facilities in Kenya and Uganda.

## Methods

### Study setting

In Kenya and Uganda, annual neonatal mortality rates have slowly decreased over the last decade, but remain high at 21 and 20 deaths per 1,000 live births, respectively (2017) [34]. Estimated preterm birth rates are 12% for Kenya and 14% for Uganda [35]. We conducted facility assessments at 23 health facilities- 17 in Migori County, Kenya and 6 in Busoga Region, Uganda. In Kenya, facilities included 1 county referral hospital, 10 sub-county hospitals, 2 mission hospitals, and 4 health centers. In Uganda, facilities included 1 regional referral hospital, 3 district hospitals, and 2 mission/private non-for-profit (PNFP) hospitals. All of the facilities were intervention sites for a larger Preterm Birth Initiative study focused on data strengthening and provider skills training.

### Study procedures

#### Cascade development

Using the WHO Guidelines for the Management of Common Childhood Illnesses [36], researchers (MM, DW) developed a list of evidence-based treatments for 6 common neonatal conditions/emergencies: essential (routine) newborn care (for all newborns); neonatal resuscitation; poor feeding-hypothermia; respiratory distress-apnea of prematurity; infection-convulsions; and jaundice. We also reviewed local guidelines, which were available for a subset of these conditions, and found them to be congruent with the WHO Guidelines [36]. Researchers (MM, HN, GMN, GN, PW, PO, DW) then developed and refined lists of essential supplies, including drugs, needed at each stage of the facility readiness cascades: identification of the condition/emergency (stage 1), treatment (stage 2), and monitoring and modifying treatment as clinically indicated (stage 3). Supplies considered infeasible for routine use in LMIC settings [IV epinephrine; X-ray machine; laboratory testing supplies (e.g., for bacterial culture, complete blood count, blood type, Coombs test); supplies for lumbar puncture and exchange transfusion)] were not included in the final cascades. Readiness was defined by the presence of all required supplies/drugs for each clinical cascade and stage of care, and overall across the 3 stages for all 6 conditions. Within each clinical cascade, readiness for individual supplies required the simultaneous presence of all preceding supplies in that cascade. Within each stage, individual supplies were organized sequentially in the order in which they would be required to to provide real-time care.

#### Facility assessments

We conducted facility assessments at two time-points, approximately nine months apart, in 2016 and 2017, to determine if any changes in supply availability occurred over time. Further, as all of the facilities were intervention sites for a larger study, it was important to monitor supply availability in order to establish any potential impacts on the ongoing project. All assessments were conducted by in-country project staff with a background in either clinical care or monitoring and evaluation. Data collectors confirmed the presence and functionality of items located in neonatal units, labor rooms, and maternity wards through visual identification. Data collectors verbally inquired with a pharmacist or other pharmacy staff member to determine the availability of drugs. Staff recorded the presence or absence of items during facility assessments using a mobile application tool on the OpenDataKit platform (https://opendatakit.org). Using this tool, data collectors could also record any additional notes of interest.

### Statistical analysis

We described facility characteristics and neonatal care variables with standard descriptive statistics, including mean, standard deviation (SD), median, interquartile range (IQR), frequency, and proportion. Point estimates for resource availability across all facilities at each time-point were summarized as counts and proportions. Changes in resource availability over time by facility were calculated using McNemar’s test. Since the dataset had fewer than 100 observations or data were not normally distributed, non-parametric statistics with two-sided tests of significance were used for all analyses. Loss of readiness was calculated by subtracting readiness at a given stage from readiness in the preceding stage. In the identification stage, readiness loss was calculated by subtracting readiness from 100%. Means were used to estimate overall readiness and loss of readiness because these measures were based on few observations, thus medians would not accurately capture the range of observations. Variability was summarized using the absolute range and SD since resource availability varied greatly. Readiness loss between stages was quantified with percentages. Since resource requirements differ based upon the expected level of care provision [8,18,37], we estimated readiness for each clinical cascade and stage of care by facility level as well as by country. In the sub-analysis of health clinics, we excluded items that are not expected to be available at this level of facility [37]. Further, we compared the readiness of facilities with a functional newborn special care unit (NSCU) to those without using the two-sample test of proportions. All statistical analyses were carried out using Stata 15.1 (StataCorp LP, College Station, Texas, United States of America).

### Ethics

We obtained ethical approval from the Institutional Review Boards of Makerere University, the Uganda National Council for Science and Technology, the Kenya Medical Research Institute, and the University of California San Francisco. The facility assessment data did not require individual informed consent.

## Results

### Facility characteristics

Among the 23 facilities assessed, the median monthly delivery volume was 52 (IQR: 29–160; Table 1). Delivery volumes were highest among facilities at the regional, district, or county level (median: 212, IQR: 188–427) and lowest among facilities at the health center level (median: 25, IQR: 21–30). Eight (35%) facilities had a functional NSCU, with a median monthly admission volume of 35 (IQR: 24–108; Table 1). Notably, 6 (100%) of Ugandan facilities had a functional NSCU relative to only 2 (12%) of Kenyan facilities.

**Table 1:** Facility characteristics by facility level.

	All facilities, N = 23	Regional/district/ county level, n = 5	Mission/PNFP level, n = 4	Sub-county level, n = 10	Health center level, n = 4
**Delivery and newborn unit admission volume**					
Monthly delivery volume, median (IQR)^a^	52 (29–160)	212 (188–427)	108 (61–145)	44 (27–63)	25 (21–30)
Functional newborn special care unit (NSCU), n (%)^b^	8 (35)	5 (100)	3 (75)	0 (0)	0 (0)
Monthly NSCU admissions, median (IQR)^c^	35 (24–108)^d^	39 (35–108)	28 (24–32)^e^	N/A	N/A
**Human resources for neonatal care**					
Pediatrician, median (IQR)	0 (0–0)	0 (0–1)	0 (0–0)	0 (0–0)	0 (0–0)
Any pediatrician, n (%)	2 (9)	2 (40)	0 (0)	0 (0)	0 (0)
General doctor, median (IQR)	1 (0–1)	1 (1–1)	3 (2–4)	0 (0–1)	0 (0–0)
Any general doctor, n (%)	12 (52)	5 (100)	4 (100)	3 (30)	0 (0.0)
Clinical officer, median (IQR)	0 (0–1)	0 (0–0)	0 (0–0)	1 (0–1)	2 (1–3)
Any clinical officer, n (%)	6 (26)	0 (0)	0 (0)	3 (30)	3 (75)
Nurse midwife, median (IQR)	8 (6–12)	14 (14–15)	10 (9–12)	7 (5–8)	6 (5–8)
Any nurse-midwife, n (%)	23 (100)	5 (100)	4 (100)	10 (100)	4 (100)

^a^ Calculated as the number of deliveries per month, averaged over 12 months (September 2016 to August 2017), by facility level.

^b^ NSCUs are expected to provide feeding support for small and sick infants (including IV fluids and nasogastric tubes); infection prevention and management (including antibiotics); oxygen therapy (with pulse oximetry); phototherapy; incubators or radiant warmers; and space for neonatal resuscitation and KMC. Tertiary facilities offering neonatal intensive care are expected to additionally provide CPAP, mechanical ventilation, surfactant therapy, and 24-hour laboratory support [8].

^c^ Calculated as the number of admissions to NSCU per month, averaged over 12 months (September 2016 to August 2017), by facility level.

^d^ Figure reflects data from all 6 Ugandan facilities and 1 of the 2 Kenyan facilities with a functional NSCU; these data are not routinely collected in Kenyan facilities below the county level.

^e^ Figure reflects data from the 2 Ugandan facilities; this data is not routinely collected in Kenyan facilities below the county level.

Across all 23 facilities, there were a median of 0 pediatricians, 1 general doctor, 0 clinical officers, and 8 nurse-midwives working in neonatal care (Table 1). Pediatricians were only available at 2 (9%) facilities, both of which were regional- or district-level hospitals in Uganda. All 6 Ugandan facilities had ≥1 general doctor working in neonatal care. Among the 17 Kenyan facilities, 6 had ≥1 general doctor, 6 had ≥1 clinical officer, and 5 had only nurse midwives working in neonatal care.

### Neonatal care resource availability

Across the 2 time-points, there was wide variability in the availability of durable goods (range: 4–91%; S1 Table) and consumable supplies (range: 17–96%; S2 Table). Availability of clean cloth or towels (-34.8%, p = 0.0325), resuscitation area with warmer (-22%, p = 0.0253), and glucometers (-30%, p = 0.0082) by facility significantly decreased over time (S1 Table). No significant changes in the availability of consumable supplies by facility were identified (S2 Table). Wide variability also existed for newborn special care tracer items [8], including oxygen (78%), pulse oximeters (22–44%), IV fluids [Ringers lactate or half normal saline/5% dextrose (91%)], and nasogastric tubes (57%; S1 and S2 Tables). The majority of facilities had dextrose (78–96%) and aminophylline (78–91%; S2 Table). A much lower proportion stocked calcium gluconate (39%) and ceftriaxone or cefotaxime (57–65%; S2 Table).

### Clinical cascade estimates of neonatal care readiness

**Fig 1:**
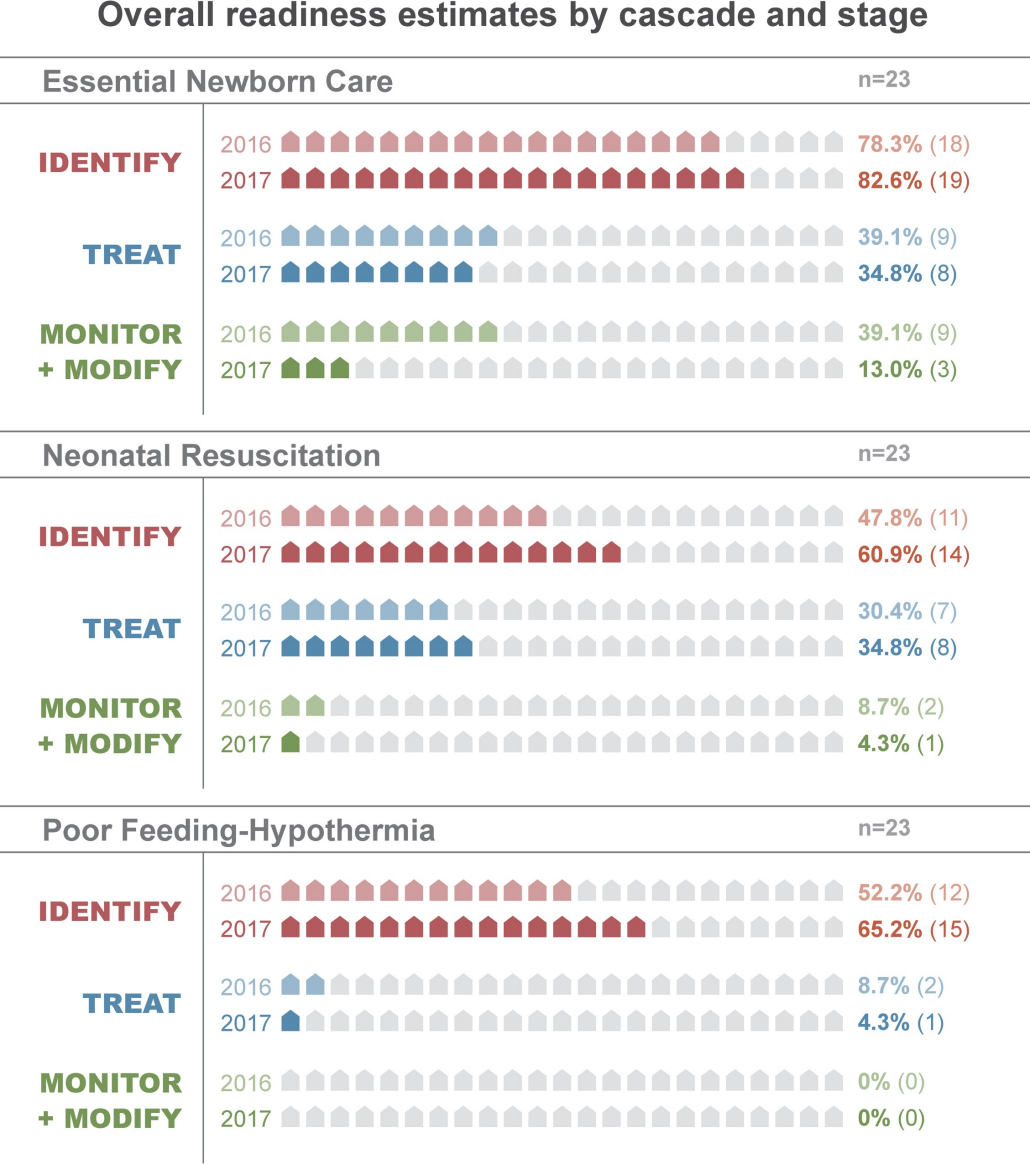
Comparison of overall readiness estimates by stage of care for the essential newborn care, neonatal resuscitation, and poor feeding-hypothermia clinical cascades in 2016 and 2017.

**Fig 2:**
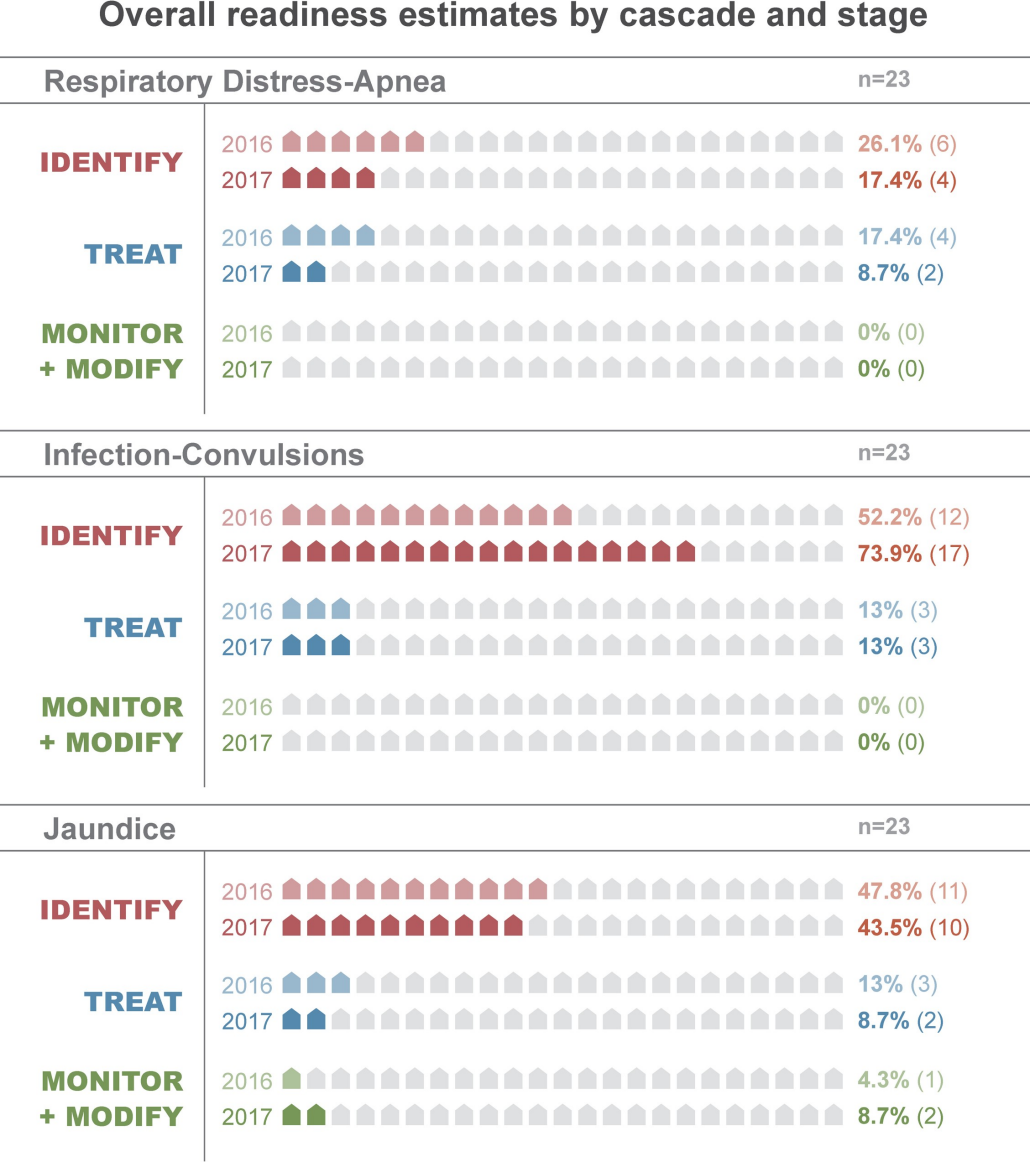
Comparison of overall readiness estimates by stage of care for the respiratory distress-apnea, infection-convulsions, and jaundice clinical cascades in 2016 and 2017.

Overall, the cascade model estimated mean readiness of 27% (SD: 23) in 2016 and 26% (SD: 28) in 2017 across the 3 stages of care for all 6 conditions. In 2016, mean readiness was 51% (SD: 17) in the identification stage, 20% (SD: 12) in the treatment stage, and 9% (SD: 15) in the monitoring-modifying stage across the 6 cascades. In 2017, mean readiness was 57% (SD: 24) in the identification stage, 17% (SD: 14) in the treatment stage, and 4% (SD: 6) in the monitoring-modifying stage. Across both time-points, mean readiness by cascade ranged from 12% (respiratory distress-apnea) to 48% (essential newborn care; Tables 2 and 3; Figs 1 and 2).

**Table 2:** Neonatal care readiness for the essential newborn care, neonatal resuscitation, and poor feeding-hypothermia clinical cascades, 2016 and 2017 (N = 23 facilities).

	Stage	Item	2016 n (%)^a^	2017 n (%)^a^
**Essential Newborn Care**	**Identify**	Water and soap, or hand disinfectant	18 (78)	19 (83)
	**Treat**	Clean blade / cord ties^b^[38]	18 (78)	17 (74)
		Vitamin K (IM)	10 (44)	8 (35)
		Tetracycline eye ointment	9 (39)	8 (35)
		PMTCT in line with national policy^c^		
	**Monitor-Modify**	Newborn weighing scale	9 (39)	6 (26)
		Guidelines: referral of sick newborns	9 (39)	3 (13)
**Neonatal Resuscitation**	**Identify**	Water and soap, or hand disinfectant	18 (78)	19 (83)
		Stethoscope	13 (57)	17 (74)
		Disposable gloves	11 (48)	14 (61)
	**Treat**	Resuscitation area with heat lamp	11 (48)	12 (52)
		Ventilation bag	9 (39)	10 (44)
		Mask–term / preterm size^d^	7 (30)	8 (35)
		Suction device	7 (30)	8 (35)
	**Monitor-Modify**	Neonatal resuscitation algorithm	3 (13)	5 (22)
		Thermometer	2 (9)	5 (22)
		Pulse oximeter with probe	2 (9)	3 (13)
		Guidelines: referral of sick newborns	2 (9)	1 (4)
**Poor Feeding- Hypothermia**	**Identify**	Water and soap, or hand disinfectant	18 (78)	19 (83)
		Newborn weighing scale	18 (78)	17 (74)
		Thermometer	17 (74)	17 (74)
		Tape measure	12 (52)	15 (65)
	**Treat**	Incubator or radiant warmer^e^ [39]	9 (39)	11 (48)
		KMC bed or chair^f^ [40]	3 (13)	6 (26)
		IV cannula sets	3 (13)	6 (26)
		IV bags or tubing	2 (9)	1 (4)
		Dextrose (IV)	2 (9)	1 (4)
		Nasogastric tube (neonatal size)	2 (9)	1 (4)
		Syringes / cups	2 (9)	1 (4)
	**Monitor-Modify**	Lancets (neonatal or infant size)	1 (4)	1 (4)
		Glucose test strips	1 (4)	1 (4)
		Glucometer	0	0
		Postnatal gestational age assessment tool^g^	0	0
		Preterm infant feeding guidelines	0	0
		Ringers lactate (in 10% dextrose) or half normal saline/ 5% dextrose^h^ [41]	0	0
		Guidelines: referral of sick newborns	0	0

^a^ For each successive item in a clinical cascade, readiness requires the simultaneous presence of all preceding items in that cascade.

^b^ Clean, dry cord care is recommended for all neonates born in health facilities. Chlorhexidine 4% is recommended only for neonates born at home in settings with high neonatal mortality (NMR ≥30), or to replace application of a harmful traditional substance to the umbilical cord (e.g., cow dung), thus it was not included.

^c^ In settings with high HIV prevalence, PMTCT is required for neonates born to mothers with positive HIV test (not assessed in this study).

^d^ Term (size 1) masks are required for normal-weight infants and preterm (size 0) masks are required for infants weighing <2500 grams (g) [36]. In this study, the presence of term or preterm size masks was assessed in facilities.

^e^ An incubator or radiant warmer is required for thermal care of neonates weighing ≤2000g who are: 1) clinically unstable, or 2) clinically stable, but mother/other caregiver is not able/available to provide KMC.

^f^ A clean cloth (may be brought by the mother), sized approximately 1 square meter, may be folded and securely tied to function as a KMC support wrap/binder. This may later be replaced by a carrying pouch of the mother’s choice.

^g^ Ballard, Dubowitz, or simplified postnatal gestational age assessment tool is required to calculate gestational age when last menstrual period (LMP) is unavailable, unreliable, or incongruent with appearance.

^h^ Ringers lactate (added to 10% dextrose in an appropriate ratio, e.g., 1:4) or half normal saline/5% dextrose is required for fluid maintenance in neonates unable to tolerate enteral feeds after the first 2 days.

**Table 3:** Neonatal care readiness for the respiratory distress-apnea, infection-convulsions, and jaundice clinical cascades, 2016 and 2017 (N = 23 facilities).

	Stage	Item	2016 n (%)^a^	2017 n (%)^a^
**Respiratory Distress-Apnea**	**Identify**	Water and soap, or hand disinfectant	18 (78)	19 (83)
		Stethoscope	13 (57)	17 (74)
		Pulse oximeter with probe	6 (26)	4 (17)
	**Treat**	Oxygen canister or concentrator	6 (26)	4 (17)
		Oxygen tubing	4 (17)	4 (17)
		Nasal cannula (neonatal size)	4 (17)	4 (17)
		Aminophylline or caffeine citrate^b^	4 (17)	3 (13)
		Ventilation bag	4 (17)	2 (9)
		Mask—term / preterm size^c^	4 (17)	2 (9)
		Suction	4 (17)	2 (9)
	**Monitor-Modify**	Guidelines: oxygen therapy^d^	2 (9)	0
		Guidelines: apnea of prematurity	0	0
		Continuous positive airway pressure (CPAP) device^e^	0	0
		Guidelines: referral of sick newborns	0	0
**Infection-Convulsions**	**Identify**	Water and soap, or hand disinfectant	18 (78)	19 (83)
		Stethoscope	13 (57)	17 (74)
		Thermometer	12 (52)	17 (74)
	**Treat**	IV cannula sets	9 (39)	10 (44)
		IV bags or tubing	3 (13)	3 (13)
		Newborn weighing scale^f^	3 (13)	3 (13)
		Ampicillin or penicillin (IV)	3 (13)	3 (13)
		Gentamicin (IV)	3 (13)	3 (13)
	**Monitor-Modify**	Guidelines: neonatal sepsis	0	2 (9)
		Lancets (neonatal or infant size)	0	2 (9)
		Glucose test strips	0	2 (9)
		Glucometer	0	0
		Dextrose (IV)	0	0
		Ceftriaxone or cefotaxime^g^	0	0
		Phenobarbital (IV)^h^		
		Calcium gluconate (IV)^h^	0	0
		Guidelines: referral of sick newborns	0	0
**Jaundice**	**Identify**	Water and soap, or hand disinfectant	18 (78)	19 (83)
		Lancets (neonatal or infant size)	11 (48)	10 (44)
		Serum bilirubin measurement or bilirubin test strips^i^ [42,43]		
	**Treat**	Phototherapy unit	3 (13)	2 (9)
		Incubator or radiant warmer^j^	3 (13)	2 (9)
	**Monitor-Modify**	Guidelines: neonatal jaundice^k^		
		Postnatal gestational age assessment tool^k^	1 (4)	2 (9)
		Newborn weighing scale^l^	1 (4)	2 (9)
		Guidelines: referral of sick newborns	1 (4)	2 (9)

^a^ For each successive item in a clinical cascade, readiness requires the simultaneous presence of all preceding items in that cascade.

^b^ Caffeine citrate (preferred) or aminophylline is required to help prevent and treat apnea in preterm infants.

^c^ Term (size 1) masks are required for normal-weight infants and preterm (size 0) masks are required for infants weighing <2500g [36]. In this study, the presence of term or preterm size masks was assessed in facilities.

^d^ Oxygen therapy guidelines are needed to help providers modify oxygen therapy based on oxygen saturation and clinical signs.

^e^ CPAP is required to provide respiratory support to infants with severe respiratory distress (in secondary/referral-level facilities).

^f^ A weighing scale is required for accurate dosing of antibiotics and other medications.

^g^ Ceftriaxone or cefotaxime is required as a second-line therapy for meningitis and other severe infections not responding to initial antibiotics within 2–3 days. Ceftriaxone is also used as a first-line therapy with tetracycline eye ointment (in Essential Newborn Care cascade) for ophthalmia neonatorum.

^h^ Phenobarbital is required to treat infants who are having convulsions (not assessed in this study). In addition, measurement of serum calcium should be considered (in facilities with laboratory capacity), with calcium gluconate 10% administered for treatment of hypocalcemia.

^i^ Bilirubin should be measured in infants with suspected hyperbilirubinemia. Serum bilirubin measurement is preferred (in facilities with laboratory capacity). Rapid bilirubin tests may be used in facilities lacking laboratory capacity (not assessed in this study).

^j^ An incubator or radiant warmer is required for thermal care of neonates weighing ≤2000g while receiving phototherapy.

^k^ Guidelines are needed to help providers assess risk of severe hyperbilirubinemia and determine treatment threshold (not assessed in this study). Ballard, Dubowitz, or other gestational age assessment tool is required to calculate gestational age (when LMP is unavailable, unreliable, or incongruent with appearance) for use in determining severe hyperbilirubinemia risk and treatment threshold.

^l^ A weighing scale is required to monitor for evidence of dehydration during phototherapy.

**Fig 3:**
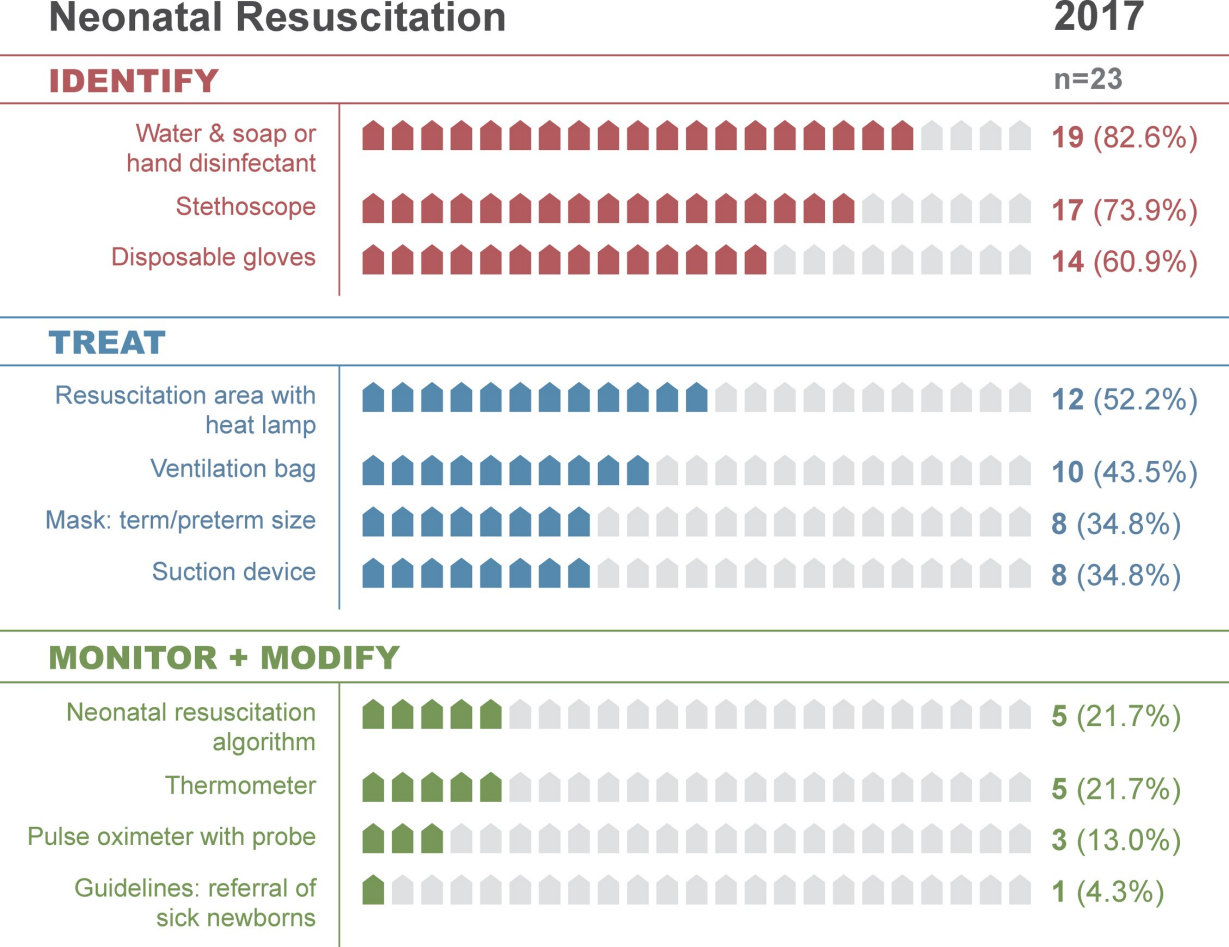
Neonatal resuscitation clinical cascade, 2017.

**Fig 4:**
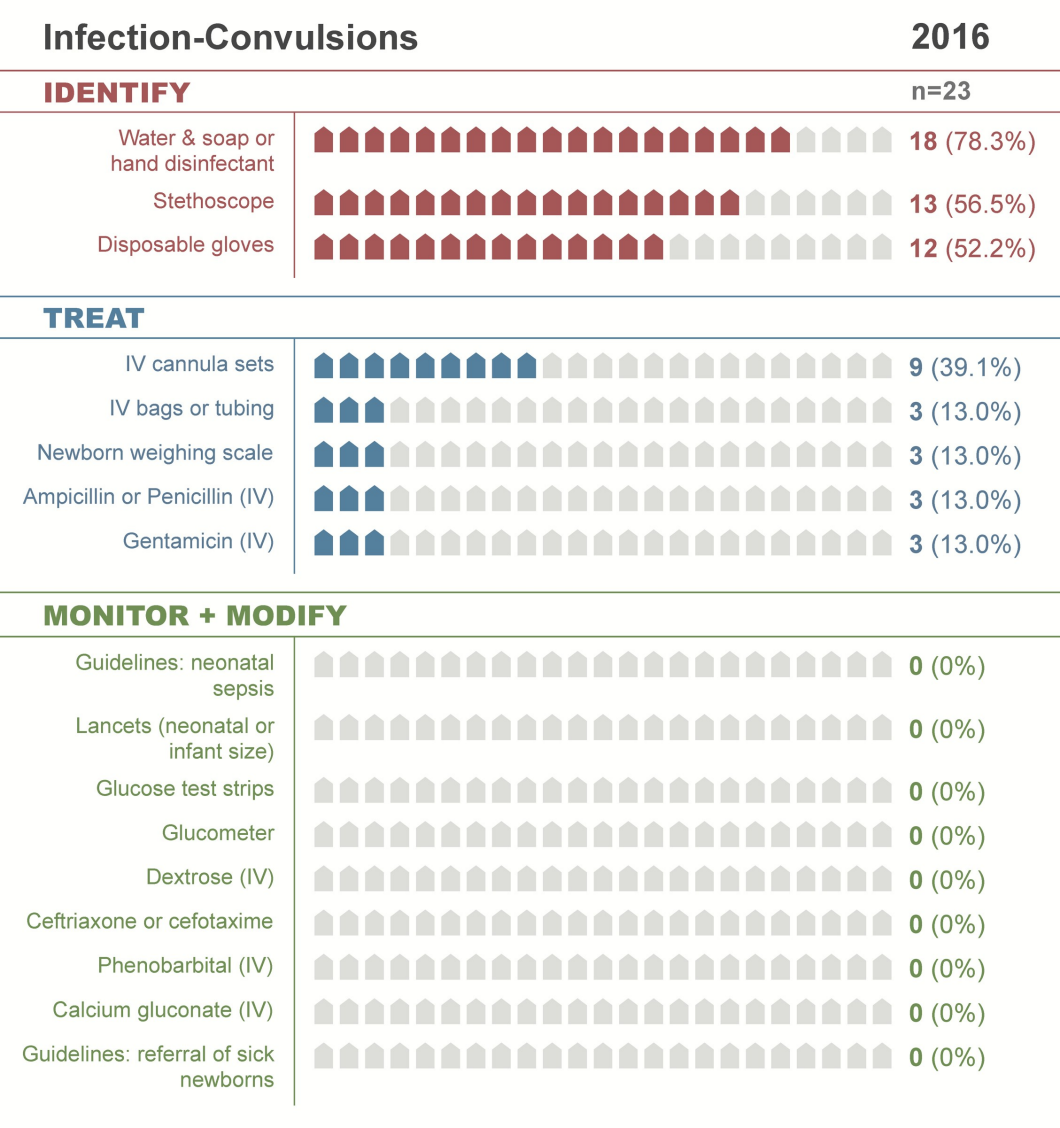
Infection-convulsions clinical cascade, 2016. See Table 3 for relevant footnotes.

In 2017, 14 of 23 facilities (61%) had the resources necessary to identify a non-vigorous neonate requiring resuscitation, including water and soap (or hand disinfectant), stethoscope, and disposable gloves (Table 2; Fig 3). Of those, 12 (52%) had a resuscitation area with heat lamp, 10 (44%) had a ventilation bag, and 8 (35%) had an appropriately-sized mask and suction device. Only 1 facility (4%) also had guidelines for referral of sick newborns, which should be present in all facilities offering newborn care as this is essential to help providers identify neonates who require a higher level of care (Table 2; Fig 3). In 2016, 12 facilities (52%) had water and soap (or disinfectant), stethoscopes, and thermometers for identifying neonatal infections (Table 3; Fig 4). Only 3 (13%) additionally had supplies to dose and administer parenteral antibiotics (IV cannula sets, IV bags or tubing, and weighing scale). Although 87% of facilities had ampicillin (or penicillin) and 78% had gentamicin (S2 Table), far fewer had the resources to identify infections and accurately administer first-line antibiotics (13% stage 2 readiness; Table 3; Figs 2 and 4).

### Clinical cascade estimates of neonatal care readiness by facility type and by country

Among the 5 regional/district/county level facilities, the cascade model estimated mean readiness of 43% (SD: 32) in 2016 and 29% (SD: 28) in 2017 across all stages and conditions (S3 Table). Overall mean readiness was 38% (SD: 31) in 2016 and 44% (SD: 37) in 2017 at the 4 mission/PNFP facilities (S3 Table). Comparatively, overall readiness was lower at the 10 sub-county level facilities [2016: mean 16 (SD: 20); 2017: mean 23 (SD: 30)] and the 4 health centers [2016: mean 19 (SD: 32); 2017: mean 27 (SD: 19); S3 Table]. Among the 17 Kenyan facilities, overall mean readiness was 23% (SD: 23) in 2016 and 22% (SD: 28) in 2017 (S4 Table). Overall mean readiness was increased at the 6 Ugandan facilities, ranging from 32% (SD: 28) in 2016 to 39% (SD: 31) in 2017 (S4 Table).

### Readiness loss by cascade

Along the cascades, there were notable differences in readiness loss from identification (stage 1) through monitoring-modifying therapy (stage 3). At both time-points, it varied least for neonatal resuscitation (range: 13–35) and most for respiratory distress-apnea (range: 65–74; Table 4; Figs 1–3; S1 Fig). There was mild to moderate variability in when readiness was lost along the cascade. In 2016, the majority of readiness was lost during the identification stage for all cascades except essential newborn care, which lost most readiness in the treatment stage (Table 4; Fig 4; S1 and S2 Figs). In 2017, the majority of readiness was again lost in the identification stage for respiratory distress-apnea, jaundice, and neonatal resuscitation (Table 4; Fig 3). In contrast, the infection-convulsions, essential newborn care, and poor feeding-hypothermia cascades lost most readiness in the treatment stage (Table 4; S3 and S4 Figs).

**Table 4:** Readiness loss by clinical cascade and stage of care, 2016 and 2017.

	Readiness loss by stage^a^			Readiness loss by cascade		
	Identify	Treat	Monitor/Modify	Mean loss across 3 stages	SD	Range
**2016**						
**Loss by clinical cascade**						
				30^b^	20	
Essential Newborn Care	22	39	0	20	20	39
Neonatal Resuscitation	52	17	22	30	19	35
Poor Feeding-Hypothermia	48	44	9	33	21	39
Respiratory Distress-Apnea	74	9	17	33	35	65
Infection-Convulsions	48	39	13	33	18	35
Jaundice	52	35	9	32	22	44
**Overall loss by stage**						
Mean loss across cascade	49	31	12			
SD	17	14	8	5		
**2017**						
**Loss by clinical cascade**						
				32^b^	23	
Essential Newborn Care	17	48	22	29	16	30
Neonatal Resuscitation	39	26	31	32	7	13
Poor Feeding-Hypothermia	35	61	4	33	28	57
Respiratory Distress-Apnea	83	9	9	33	43	74
Infection-Convulsions	26	61	13	33	25	48
Jaundice	57	35	0	30	29	57
**Overall loss by stage**						
Mean loss across cascade	43	40	13			
SD	24	21	11	2		

^a^ n = 23 facilities

^b^ This figure represents overall mean readiness loss across the 3 stages for all 6 cascades.

### Readiness loss by stage

Across all 6 cascades, mean readiness loss by stage ranged from 43–49% for identification, 31–40% for treatment, and 12–13% for monitoring-modifying treatment (Table 4). Across all cascades and stages, there was an increasingly consistent pattern of 30% (SD: 5) to 32% (SD: 2) overall readiness loss, with moderate variability in how loss occurred across stages (SD across stages: 20–23; Table 4).

### Comparison of readiness in facilities with and without newborn special care units

Across both time-points, overall mean readiness was higher among facilities with a functional NSCU (37%) compared to those without (20%). For both groups, readiness was again lowest for respiratory distress-apnea (27% and 3%, respectively) and highest for essential newborn care (54% and 39%, respectively; Table 5). Among facilities with a NSCU, there was significantly increased identification readiness for respiratory distress-apnea (2016: 63% vs. 7%; 2017: 38% vs. 7%) and jaundice (2016: 75% vs. 33%), and treatment readiness for essential newborn care (2016: 75% vs. 20%), poor feeding-hypothermia (2016: 25% vs. 0%), respiratory distress-apnea (2016: 38% vs. 7%; 2017: 25% vs. 0%), and infection-convulsions (2017: 38% vs. 0%; Table 5). At the monitoring-modifying stage, a significant difference was only identified for essential newborn care (2016: 38% vs. 7%; Table 5).

**Table 5:** Comparison of readiness in facilities with and without newborn special care units, 2016 and 2017.

Stage	Neonatal care cascade	2016 time-point			2017 time-point		
		NSCU present^a^	NSCU absent^b^	p-value^c^	NSCU present^a^	NSCU absent^b^	p-value^c^
**Identify**	Essential Newborn Care (n, %)	7 (88)	11 (73)	0.2159	7 (88)	12 (80)	0.3256
	Neonatal Resuscitation (n, %)	4 (50)	7 (47)	0.4400	6 (75)	8 (53)	0.1549
	Poor Feeding-Hypothermia (n, %)	6 (75)	6 (40)	0.0548	6 (75)	9 (60)	0.2360
	Respiratory Distress-Apnea (n, %)	5 (63)	1 (7)	0.0019	3 (38)	1 (7)	0.0318
	Infection-Convulsions (n, %)	5 (63)	7 (47)	0.2350	7 (88)	10 (67)	0.1396
	Jaundice (n, %)	6 (75)	5 (33)	0.0283	5 (63)	5 (33)	0.0892
**Treat**	Essential Newborn Care (n, %)	6 (75)	3 (20)	0.0050	3 (38)	5 (33)	0.4202
	Neonatal Resuscitation (n, %)	3 (38)	4 (27)	0.2960	4 (50)	4 (27)	0.1319
	Poor Feeding-Hypothermia (n, %)	2 (25)	0 (0)	0.0214	1 (13)	0 (0)	0.0807
	Respiratory Distress-Apnea (n, %)	3 (38)	1 (7)	0.0318	2 (25)	0 (0)	0.0214
	Infection-Convulsions (n, %)	2 (25)	1 (7)	0.1074	3 (38)	0 (0)	0.0055
	Jaundice (n, %)	2 (25)	1 (7)	0.1074	1 (13)	1 (7)	0.9936
**Monitor-Modify**	Essential Newborn Care (n, %)	3 (38)	1 (7)	0.0318	0 (0.0)	3 (20.0)	0.9125
	Neonatal Resuscitation (n, %)	1 (13)	1 (7)	0.3193	1 (13)	0 (0)	0.0807
	Poor Feeding-Hypothermia (n, %)	0 (0)	0 (0)	1.0000	0 (0)	0 (0)	1.0000
	Respiratory Distress-Apnea (n, %)	0 (0)	0 (0)	1.0000	0 (0)	0 (0)	1.0000
	Infection-Convulsions (n, %)	0 (0)	0 (0)	1.0000	0 (0)	0 (0)	1.0000
	Jaundice (n, %)	1 (13)	0 (0)	0.0807	1 (13)	1 (7)	0.9936

^a^ Facilities with functional NSCU, n = 8

^b^ Facilities without functional NSCU, n = 15

^c^ p-values were calculated using the two-sample test of proportions, with a 95% level of confidence.

## Discussion

The clinical cascade model offers a novel, stepwise approach to quantitatively estimate facility readiness for neonatal care in LMICs. Cascade-derived indicators, including overall readiness, readiness loss by cascade, readiness loss by stage, and aggregate readiness loss, can be used by health administrators, policy-makers, program managers, and researchers to assess and monitor the availability of drugs, supplies, and equipment for facility-based neonatal care in such contexts. By precisely identifying the timing and location of readiness loss, by stage or clinical condition, the cascades could help guide resource allocation decisions and facilitate provision of available, evidence-based interventions to reduce neonatal morbidity and mortality [7]. Further, aggregate readiness loss may be utilized to evaluate and compare readiness for neonatal care across health systems, countries, or geographic regions [33].

In contrast to health facility inventories and signal functions widely used to evaluate EmONC capacity [16,44,45], the cascades pragmatically assess and quantify a facility’s capacity to identify and manage six common neonatal conditions. This is accomplished by modeling the hierarchical and interdependent relationship among the resources required to identify a condition, provide initial treatment, monitor clinical response, and modify treatment if indicated [24,25]. Although additional signal functions for emergency newborn care were proposed in 2012 [18], these have not been incorporated in recent WHO guidelines [38,39]. Further, a standardized approach to evaluate readiness for basic newborn care using existing indicators is lacking [16,33].

To help address this gap, the cascade model provides an intuitive set of overall, condition-specific, and health system readiness indicators for basic and comprehensive levels of newborn care. Notably, this approach entails a negligible increase in data collection requirements compared to existing facility assessment inventories [44,45]. In addition, the neonatal cascades provide detailed information about when and where readiness loss occurs. Aggregate readiness loss can be used as a standardized indicator to quantify and compare readiness at the facility-, health system-, or national-level. This study identified a consistent pattern of 30–32% overall readiness loss across cascades and stages, which is comparable to that seen in the obstetric cascade study in Kenya [33]. The majority of readiness loss occurred in the identification stage; however, loss of treatment readiness increased in 2017, with this stage accounting for the majority of overall loss for essential newborn care, poor feeding-hypothermia, and infection-convulsions. Comparatively, the obstetric cascade study found increased variability in timing of readiness loss. For example, most loss for hypertensive emergencies occurred in the identification stage, whereas most loss for hemorrhage occurred in the monitoring-modifying stage [33].

Using the cascade model, we found that overall readiness for neonatal care was 26% among the 23 facilities at both time-points. In comparison, three studies using the EmONC signal function classification to assess a total of 431 facilities in sub-Saharan Africa and South Asia found that 0–9% and 0–23% were able to provide basic and comprehensive levels of care, respectively [19–21]. Readiness was consistently highest for essential newborn care. This may be related to the fact that resources required for comprehensive neonatal care are more expensive or difficult to maintain than those needed for essential newborn care. Additionally, previous studies evaluating facility or health system capacity have largely focused on indicators related to basic newborn care, including cleanliness, breastfeeding, cord care, tetracycline eye ointment, vitamin K, and resuscitation at birth [18–22,46–48]. Conversely, readiness was consistently lowest for respiratory distress-apnea, with only 17–26% of facilities having a pulse oximeter and other supplies required for identification. Two previous studies in Kenya similarly found that 14–18% of public referral hospitals had functional pulse oximeters for pediatric and neonatal care in 2012 [49,50]. Not surprisingly, overall readiness was higher in regional/district/county level and mission/PNFP facilities relative to sub-county and health clinic level facilities. In line with the WHO guidelines on managing possible serious bacterial infections in young infants when referral is not feasible [51], we replaced IV cannula sets with sterile syringes and needles (for IM injection) in the neonatal infection-convulsions cascade sub-analysis of health clinics. By country, overall readiness was higher in Uganda (32–39%) than in Kenya (22–23%). This is likely related to the fact that 100% of Ugandan facilities had a functional NSCU, whereas nearly 90% of Kenyan facilities did not.

Endorsed in 2014, the Every Newborn Action Plan (ENAP) is a global multi-partner initiative to prevent stillbirths and reduce neonatal mortality, with national targets of 10 or fewer deaths per 1000 livebirths by 2035 [52]. To help improve the provision of facility-based care, ENAP has recommended defining indicators for intervention packages by level of care (basic, special, or intensive care), noting that many small and sick neonates can be appropriately managed in NSCUs [3,8,23]. A Delphi study suggested that special care, including KMC, feeding support, IV fluids, oxygen, and management of infections and jaundice, could prevent 70% of deaths in preterm neonates [7]. We identified increased overall readiness among facilities with a NSCU compared to those without. Further, facilities with a NSCU had significantly increased treatment readiness for essential newborn care, poor feeding-hypothermia, respiratory distress-apnea, and infection-convulsions, relative to facilities without a NSCU. Recognizing the need to improve care for small and sick neonates, the Government of India scaled-up the establishment of NSCUs in district hospitals across the country [53–55]. A study of eight NSCUs in eight Indian states, all established within the preceding five years, demonstrated that cause-specific mortality due to sepsis and low birthweight decreased significantly over a two-year period [55].

Notably, poor-quality care is now considered to be a greater barrier to mortality reduction in LMICs than insufficient access [56]. The WHO has developed a quality of care framework for pregnant women and newborns in facilities, which highlights the overarching need for both competent human resources and essential physical resources and additionally requires evidence-based practices for routine and emergency care; actionable information systems; functional referral systems; effective communication; respect and dignity; and emotional support [57]. In recent years, increased emphasis has been placed on promotion of respectful maternity care and elimination of abuse during childbirth [58–60]. One study found that women who experience discrimination or abuse during childbirth are less likely to seek facility-based delivery care in the future [61]; such experiences may also deter postnatal care-seeking [12]. To help improve clinical outcomes for small and sick newborns, a culture of capability should be promoted in places where fatalism on the part of healthcare providers is common [62,63]. Evidence from high- and middle-income countries has also demonstrated the value of family-centered developmental care for this vulnerable population [64–66]. A study in Colombia found that continuing education for care providers, provision of materials for positioning of neonates, and use of an informative video for parents were helpful in promoting related care practices [67].

This study has several limitations. The cascade model assesses the physical readiness of facilities to provide newborn care, but it does not assess human resource availability or healthcare providers’ skills. Observational data from 18 LMICs, including Kenya and Uganda, showed that providers fulfilled 45% and 64% of recommended elements of sick child and delivery care, respectively [56], highlighting the fact that provider skill assessment is also imperative. These data are from 23 facilities in two regions within two countries of East Africa, which limits generalizability to all LMIC contexts. All facility assessments were routinely conducted as part of a broader maternal and newborn health research initiative. As a result, a few variables necessary for complete modeling of the essential newborn care, infection-convulsions, and jaundice cascades were not available. Significant and unanticipated reductions in the availability of certain durable goods (e.g., resuscitation area with warmer, glucometer) were identified; however, we did not obtain data about potential reasons why these items were no longer present (or functional). Certain tracer items for basic and special levels of newborn care are poorly defined, which may slightly limit comparisons of this study with previous studies using these indicators from the literature. For example, the EmONC tracer for basic neonatal resuscitation does not specify the ventilation bag or mask size [16], and the ENAP tracer for IV fluid does not specify the type of fluid [8]. Clearly defined tracer items are imperative to standardize readiness estimates for neonatal care and promote comparability across study results and settings.

In the future, research should evaluate the neonatal cascade model in a variety of cultural, regional, and national contexts. In addition, studies comparing this novel model of neonatal care readiness with previous models may be indicated. Notably, the third stage (monitoring-modifying treatment) of each cascade includes one or more guidelines related to newborn care practices, e.g., referral of sick newborns; however, few previous studies assessing facility readiness for EmONC have utilized clinical guidelines as tracer items [33,47,68]. To assess the quality of facility-based neonatal care and compare readiness estimates using different models, inclusion of tracers for key clinical guidelines is essential. Finally, research could evaluate the ability of the cascades, employed as one component of a broader model, to predict neonatal mortality and morbidities related to the 6 conditions and explore the association between aggregate readiness loss and neonatal mortality across countries or regions.

## Conclusion

In conclusion, the clinical cascade model provides a novel, stepwise approach to quantitatively assess facility readiness for neonatal care. We identified a consistent pattern of 30–32% readiness loss across cascades and stages at both time-points at 23 facilities in Kenya and Uganda. This aggregate measure could be used to monitor and compare readiness at the facility-, health system-, or national-level. Cascade-derived estimates of readiness and capacity loss may help guide strategies to improve care, prioritize resources, and promote neonatal survival in LMICs.

## Supporting information

S1 Table Frequency and proportion of facilities with durable goods for neonatal care. (DOCX)

S2 Table Frequency and proportion of facilities with consumable supplies for neonatal care. (DOCX)

S3 Table Neonatal care readiness in Kenyan and Ugandan health facilities by facility level, 2016 and 2017. (DOCX)

S4 Table Neonatal care readiness in Kenyan and Ugandan health facilities by country, 2016 and 2017. (DOCX)

S1 Fig Respiratory distress-apnea clinical cascade, 2016. See Table 3 for relevant footnotes.
(TIFF)

S2 Fig Jaundice clinical cascade, 2016. See Table 3 for relevant footnotes.
(TIFF)

S3 Fig Essential newborn care clinical cascade, 2017. See Table 2 for relevant footnotes.
(TIFF)

S4 Fig Poor feeding-hypothermia clinical cascade, 2017. See Table 2 for relevant footnotes.
(TIFF)
