# Supplementary material for: Clinical cascades as a novel way to assess physical readiness of facilities for the care of small and sick neonates in Kenya and Uganda
Source: PLoS One. 2018 Nov 21;13(11):e0207156. doi: 10.1371/journal.pone.0207156 (PMC6248954; doi:10.1371/journal.pone.0207156)
Supplement: S1 Table — (DOCX) [file pone.0207156.s001.docx]

**S1 Table. Frequency and proportion of facilities with durable goods for neonatal care**

| **Category** | **Item** | **2016**  **n (%)^a^** | **2017**  **n (%)^a^** | **Percentage point change** | **p-value^b^** |
| --- | --- | --- | --- | --- | --- |
| **Physical exam supplies** | Thermometer | 21 (91) | 21 (91) | 0 | 1.0000 |
|  | Stethoscope | 15 (65) | 21 (91) | 26 | 0.0578 |
|  | Functional pulse oximeter with probe | 10 (44) | 5 (22) | -22 | 0.0588 |
|  | Tape measure | 16 (70) | 21 (91) | 21 | 0.0956 |
|  | Newborn weighing scale | 23 (100) | 21 (91) | -9 | 0.1573 |
| **Testing and treatment supplies** | Clean blade | 22 (96) | 19 (83) | -13 | 0.1797 |
|  | Clean cloth / towel (may be brought by mother) | 12 (52) | 4 (17) | -35 | 0.0325 |
|  | Resuscitation area with warmer / heat lamp | 22 (96) | 17 (74) | -22 | 0.0253 |
|  | Neonatal ventilation bag | 18 (78) | 15 (65) | -13 | 0.2568 |
|  | Mask – term or preterm size | 15 (65) | 17 (74) | 9 | 0.5637 |
|  | Oxygen tubing | 15 (65) | 15 (65) | 0 | 1.0000 |
|  | Suction device | 21 (91) | 20 (87) | -4 | 0.6547 |
|  | Glucometer | 8 (35) | 1 (4) | -31 | 0.0082 |
|  | Vitamin K (IM) | 13 (57) | 9 (39) | -18 | 0.2059 |
| **Equipment** | Functional incubator or radiant warmer | 19 (83) | 15 (65) | -18 | 0.1025 |
|  | KMC beds or chairs | 6 (26) | 7 (30) | 4 | 0.7055 |
|  | Filled oxygen cylinder or functional concentrator | 18 (78) | 18 (78) | 0 | 1.0000 |
|  | Continuous positive airway pressure (CPAP) device | 10 (44) | 5 (22) | -22 | 0.0956 |
|  | Functional phototherapy unit | 6 (26) | 7 (30) | 4 | 0.6547 |
| **Guidelines-assessment and treatment** | Guidelines: referral of sick newborns | 6 (26) | 10 (44) | 18 | 0.2059 |
|  | Neonatal resuscitation algorithm | 7 (30) | 15 (65) | 35 | 0.0209 |
|  | Gestational age assessment tool | 3 (13) | 8 (35) | 22 | 0.0588 |
|  | Preterm infant fluid / feeding guidelines | 6 (26) | 7 (30) | 4 | 0.6547 |
|  | Guidelines: oxygen therapy | 3 (13) | 6 (26) | 13 | 0.2568 |
|  | Guidelines: apnea of prematurity | 1 (4) | 9 (39) | 35 | 0.0047 |
|  | Guidelines: treatment of neonatal sepsis | 3 (13) | 9 (39) | 26 | 0.0339 |
|  | Guidelines: neonatal jaundice^c^ |  |  |  |  |

^a^ Data represent resource availability across all 23 health facilities at one time-point.

^b^ Individual facilities were paired and p-values for resource availability by facility were calculated using McNemar’s test.

^c^ Presence of guidelines for neonatal jaundice was not assessed in this study.
