# Supplementary material for: Clinical cascades as a novel way to assess physical readiness of facilities for the care of small and sick neonates in Kenya and Uganda
Source: PLoS One. 2018 Nov 21;13(11):e0207156. doi: 10.1371/journal.pone.0207156 (PMC6248954; doi:10.1371/journal.pone.0207156)
Supplement: S2 Table — (DOCX) [file pone.0207156.s002.docx]

**S2 Table. Frequency and proportion of facilities with consumable supplies for neonatal care**

| **Stage** | **Item** | **2016**  **n (%)^a^** | **2017**  **n (%)^a^** | **Percentage point change** | **p-value^b^** |
| --- | --- | --- | --- | --- | --- |
| **Examination and testing supplies** | Water & soap (or hand disinfectant) | 18 (78) | 19 (83) | 5 | 0.7055 |
|  | Disposable gloves | 17 (74) | 19 (83) | 9 | 0.4142 |
|  | Lancets (neonatal or infant size) | 13 (57) | 11 (48) | -9 | 0.5271 |
|  | Glucose test strips | 7 (30) | 4 (17) | -13 | 0.2568 |
|  | Serum bilirubin measurement or bilirubin test strips^c^ |  |  |  |  |
| **Medications** | Tetracycline eye ointment | 16 (70) | 17 (74) | 4 | 0.7055 |
|  | PMTCT in line with national policy^c^ |  |  |  |  |
|  | Dextrose (IV) | 22 (96) | 18 (78) | -18 | 0.1025 |
|  | Ringers lactate or half normal saline/5% dextrose | 21 (91) | 21 (91) | 0 | 1.0000 |
|  | Caffeine citrate or aminophylline | 21 (91) | 18 (78) | -13 | 0.2568 |
|  | Ampicillin or penicillin (IV) | 20 (87) | 14 (61) | -26 | 0.0578 |
|  | Gentamicin (IV) | 18 (78) | 17 (74) | -4 | 0.7389 |
|  | Ceftriaxone or cefotaxime (IV) | 13 (57) | 15 (65) | 8 | 0.5271 |
|  | Phenobarbital (IV)^c^ |  |  |  |  |
|  | Calcium gluconate (IV) | 9 (39) | 9 (39) | 0 | 1.0000 |
| **Other treatment supplies** | IV cannula sets (minimum size: 24 gauge) | 17 (74) | 15 (65) | -9 | 0.4795 |
|  | IV bags / tubing | 6 (26) | 4 (17) | -9 | 0.3173 |
|  | Nasogastric tube (neonatal size) | 13 (57) | 13 (57) | 0 | 1.0000 |
|  | Syringes / feeding cups | 19 (83) | 20 (87) | 4 | 0.6547 |
|  | Nasal cannula (neonatal size) | 12 (52) | 10 (44) | -8 | 0.5930 |

^a^ Data represent resource availability across all 23 health facilities at one time-point.

^b^ Individual facilities were paired and p-values for resource availability by facility were calculated using McNemar’s test.

^c^ Presence of these supplies and medications was not assessed in this study.
