# Supplementary material for: Clinical cascades as a novel way to assess physical readiness of facilities for the care of small and sick neonates in Kenya and Uganda
Source: PLoS One. 2018 Nov 21;13(11):e0207156. doi: 10.1371/journal.pone.0207156 (PMC6248954; doi:10.1371/journal.pone.0207156)
Supplement: S3 Table — (DOCX) [file pone.0207156.s003.docx]

**S3 Table. Neonatal care readiness in Kenyan and Ugandan health facilities by facility level, 2016 and 2017**

| **Clinical cascade** | **Stage** | **Regional/district/ county level, n=5** | | **Mission/PNFP level, n=4** | | **Sub-county level, n=10** | | **Health center level, n=4** | |
| --- | --- | --- | --- | --- | --- | --- | --- | --- | --- |
|  |  | **2016**  **n (%)** | **2017**  **n (%)** | **2016**  **n (%)** | **2017**  **n (%)** | **2016**  **n (%)** | **2017**  **n (%)** | **2016**  **n (%)** | **2017**  **n (%)** |
| Essential Newborn Care | Identify | 5 (100) | 4 (80) | 3 (75) | 4 (100) | 6 (60) | 9 (90) | 4 (100) | 2 (50) |
|  | Treat | 4 (80) | 1 (20) | 3 (75) | 3 (75) | 2 (20) | 3 (30) | 0 | 1 (25) |
|  | Monitor-Modify | 2 (40) | 0 | 2 (50) | 1 (25) | 0 | 1 (10) | 0 | 1 (25) |
| Neonatal Resuscitation | Identify | 2 (40) | 3 (60) | 3 (75) | 3 (75) | 4 (40) | 6 (60) | 2 (50) | 2 (50) |
|  | Treat | 2 (40) | 2 (40) | 2 (50) | 2 (50) | 3 (30) | 3 (30) | 0 | 1 (25) |
|  | Monitor-Modify | 1 (20) | 1 (20) | 1 (25) | 0 | 0 | 0 | 0 | 0 |
| Poor Feeding- Hypothermia | Identify | 5 (100) | 3 (60) | 2 (50) | 4 (100) | 3 (30) | 6 (60) | 2 (50) | 2 (50) |
|  | Treat | 2 (40) | 1 (20) | 0 | 0 | 0 | 0 |  | |
|  | Monitor-Modify | 0 | 0 | 0 | 0 | 0 | 0 | 0^a^ | 2 (50)^a^ |
| Respiratory Distress-Apnea | Identify | 3 (60) | 1 (20) | 3 (75) | 2 (50) | 0 | 1 (10) | 0 | 0 |
|  | Treat | 2 (40) | 0 | 2 (50) | 2 (50) | 0 | 0 | 0^b^ | 0^b^ |
|  | Monitor-Modify | 0 | 0 | 0 | 0 | 0 | 0 | 0^a^ | 0^a^ |
| Infection-Convulsions | Identify | 3 (60) | 4 (80) | 3 (75) | 3 (75) | 3 (30) | 8 (80) | 3 (75) | 2 (50) |
|  | Treat | 1 (20) | 1 (20) | 1 (25) | 2 (50) | 1 (10) | 0 | 1 (25)^c^ | 1 (25)^c^ |
|  | Monitor-Modify | 0 | 0 | 0 | 0 | 0 | 0 | 0^a^ | 1 (25)^a^ |
| Jaundice | Identify | 4 (80) | 3 (60) | 2 (50) | 3 (75) | 5 (50) | 3 (30) | 0 | 1 (25) |
|  | Treat | 2 (40) | 1 (20) | 0 | 3 (75) | 1 (10) | 1 (10) |  | |
|  | Monitor-Modify | 1 (20) | 1 (20) | 0 | 0 | 0 | 1 (10) | 0^d^ | 1 (25)^d^ |

^a^ Modified to include only guidelines for referral of sick newborns.

^b^ Modified to include only mask, ventilation bag, and suction.

^c^ Modified to include only newborn weighing scale, ampicillin (or penicillin) and gentamicin, with addition of sterile syringe and 23-25 gauge needle (for intramuscular injection)

^d^ Modified to include only newborn weighing scale and guidelines for referral of sick newborns.
