# Supplementary material for: Clinical cascades as a novel way to assess physical readiness of facilities for the care of small and sick neonates in Kenya and Uganda
Source: PLoS One. 2018 Nov 21;13(11):e0207156. doi: 10.1371/journal.pone.0207156 (PMC6248954; doi:10.1371/journal.pone.0207156)
Supplement: S4 Table — (DOCX) [file pone.0207156.s004.docx]

**S4 Table. Neonatal care readiness in Kenyan and Ugandan health facilities by country, 2016 and 2017**

| **Clinical cascade** | **Stage of care** | **Kenyan facilities, n=17** | | **Ugandan facilities, n=6** | |
| --- | --- | --- | --- | --- | --- |
|  |  | **2016**  **n (%)** | **2017**  **n (%)** | **2016**  **n (%)** | **2017**  **n (%)** |
| Essential Newborn Care | Identify | 13 (76) | 14 (82) | 5 (83) | 5 (83) |
|  | Treat | 5 (29) | 5 (29) | 4 (67) | 3 (50) |
|  | Monitor-Modify | 3 (18) | 3 (18) | 1 (17) | 0 |
| Neonatal Resuscitation | Identify | 9 (53) | 10 (59) | 2 (33) | 4 (67) |
|  | Treat | 6 (35) | 4 (24) | 1 (17) | 4 (67) |
|  | Monitor-Modify | 2 (12) | 0 | 0 | 1 (17) |
| Poor Feeding- Hypothermia | Identify | 7 (41) | 11 (65) | 5 (83) | 4 (67) |
|  | Treat | 0 | 0 | 2 (33) | 1 (17) |
|  | Monitor-Modify | 0 | 0 | 0 | 0 |
| Respiratory Distress-Apnea | Identify | 3 (18) | 1 (6) | 3 (50) | 3 (50) |
|  | Treat | 3 (18) | 0 | 1 (17) | 2 (33) |
|  | Monitor-Modify | 0 | 0 | 0 | 0 |
| Infection-Convulsions | Identify | 9 (53) | 12 (71) | 3 (50) | 5 (83) |
|  | Treat | 1 (6) | 0 | 2 (33) | 3 (50) |
|  | Monitor-Modify | 0 | 0 | 0 | 0 |
| Jaundice | Identify | 7 (41) | 5 (29) | 4 (67) | 5 (83) |
|  | Treat | 2 (12) | 1 (6) | 1 (17) | 1 (17) |
|  | Monitor-Modify | 0 | 1 (6) | 1 (17) | 1 (17) |
