# Supplementary figures and images for: Clinical cascades as a novel way to assess physical readiness of facilities for the care of small and sick neonates in Kenya and Uganda
Source: PLoS One. 2018 Nov 21;13(11):e0207156. doi: 10.1371/journal.pone.0207156 (PMC6248954; doi:10.1371/journal.pone.0207156)

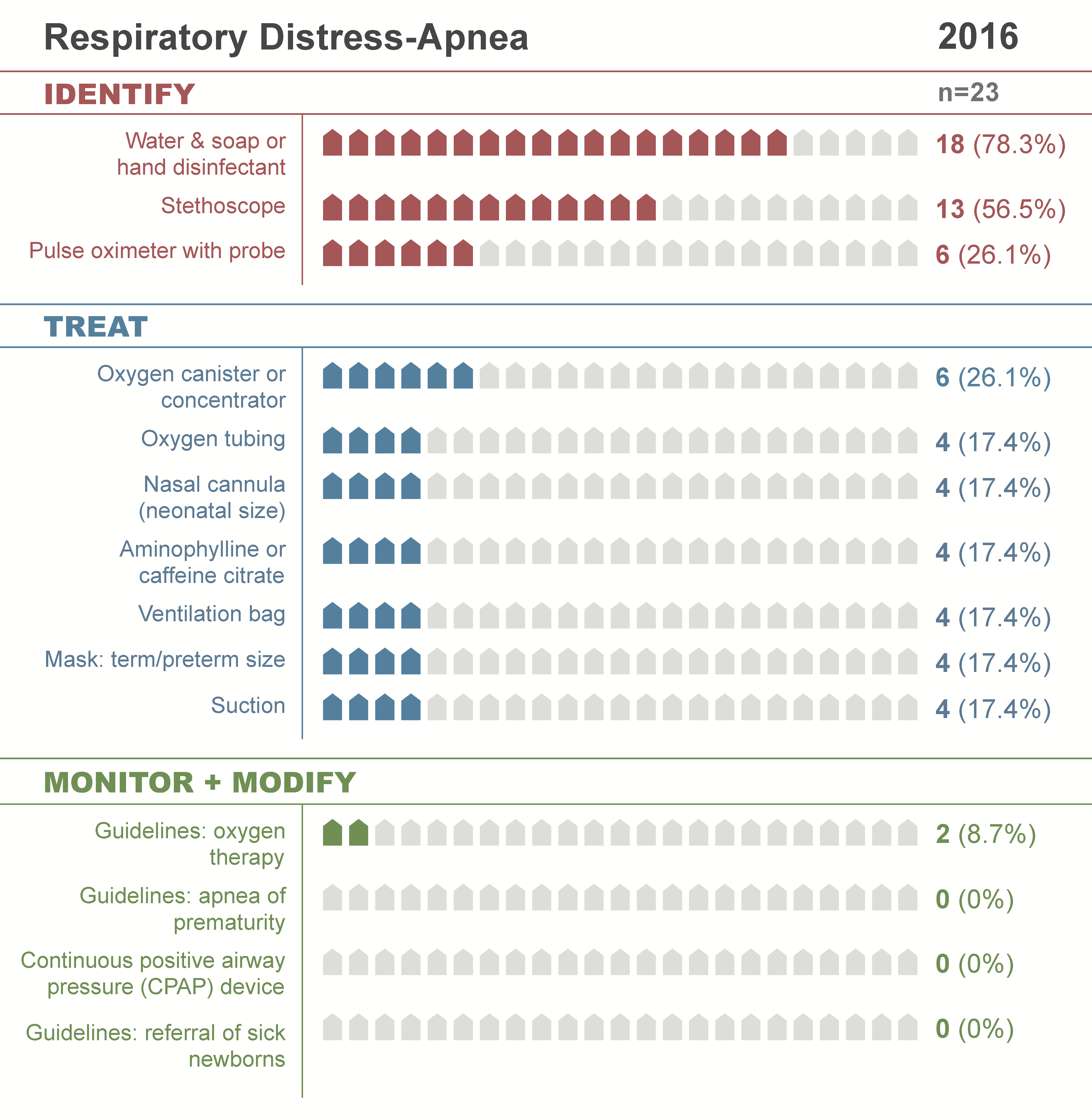

Supplement: S1 Fig — See Table 3 for relevant footnotes. (TIFF) [file pone.0207156.s005.tiff]

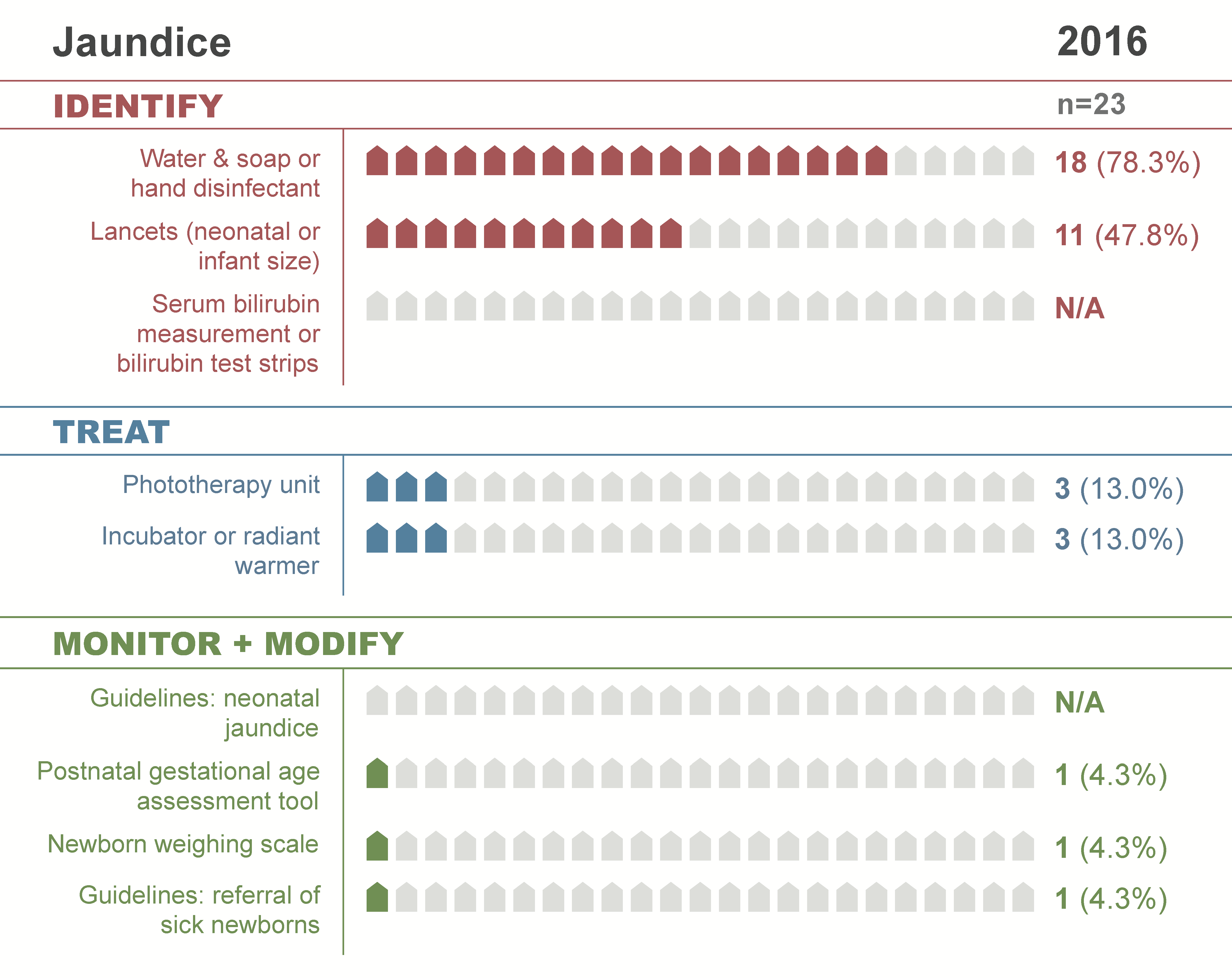

Supplement: S2 Fig — See Table 3 for relevant footnotes. (TIFF) [file pone.0207156.s006.tiff]

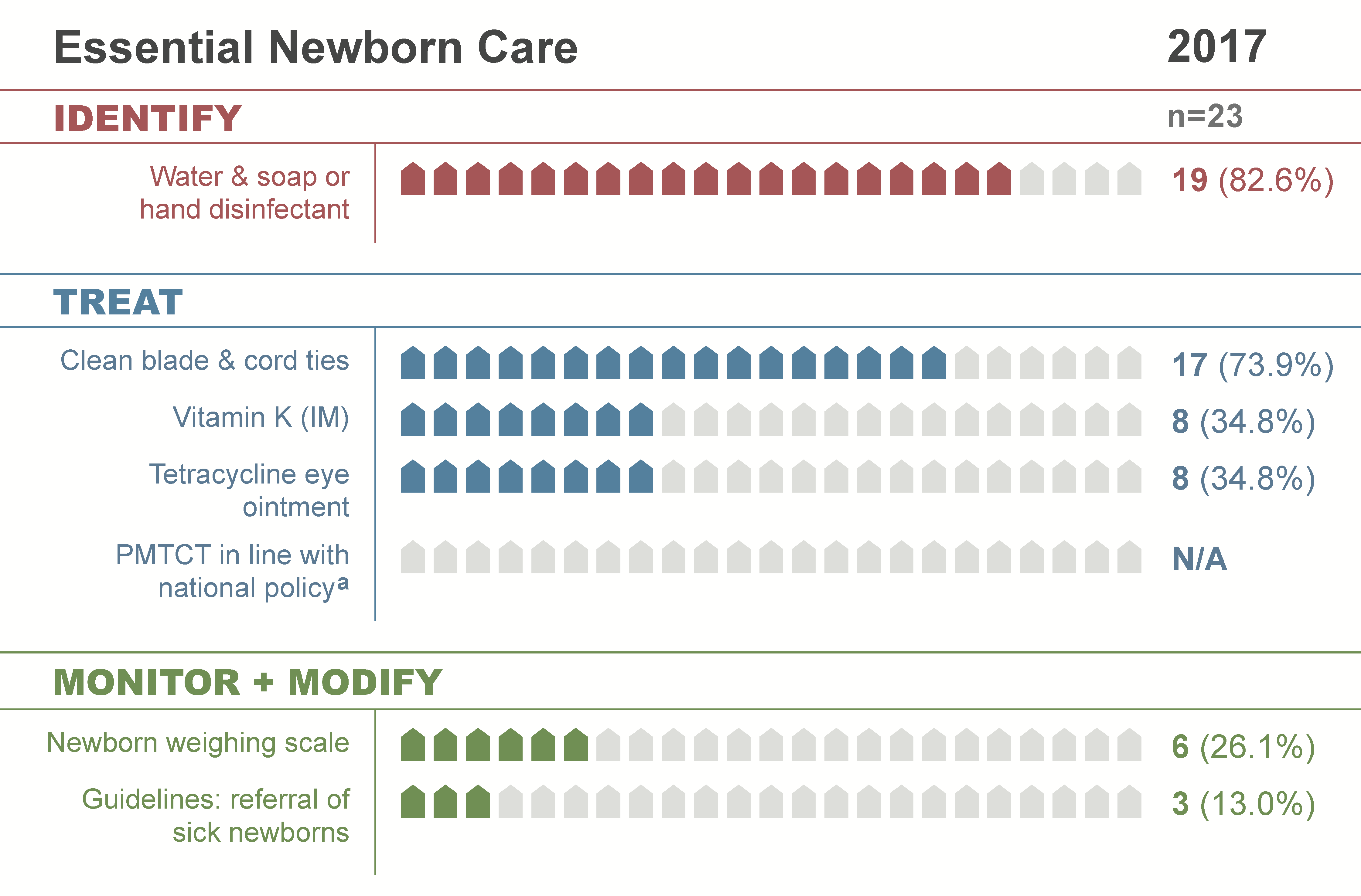

Supplement: S3 Fig — See Table 2 for relevant footnotes. (TIFF) [file pone.0207156.s007.tiff]

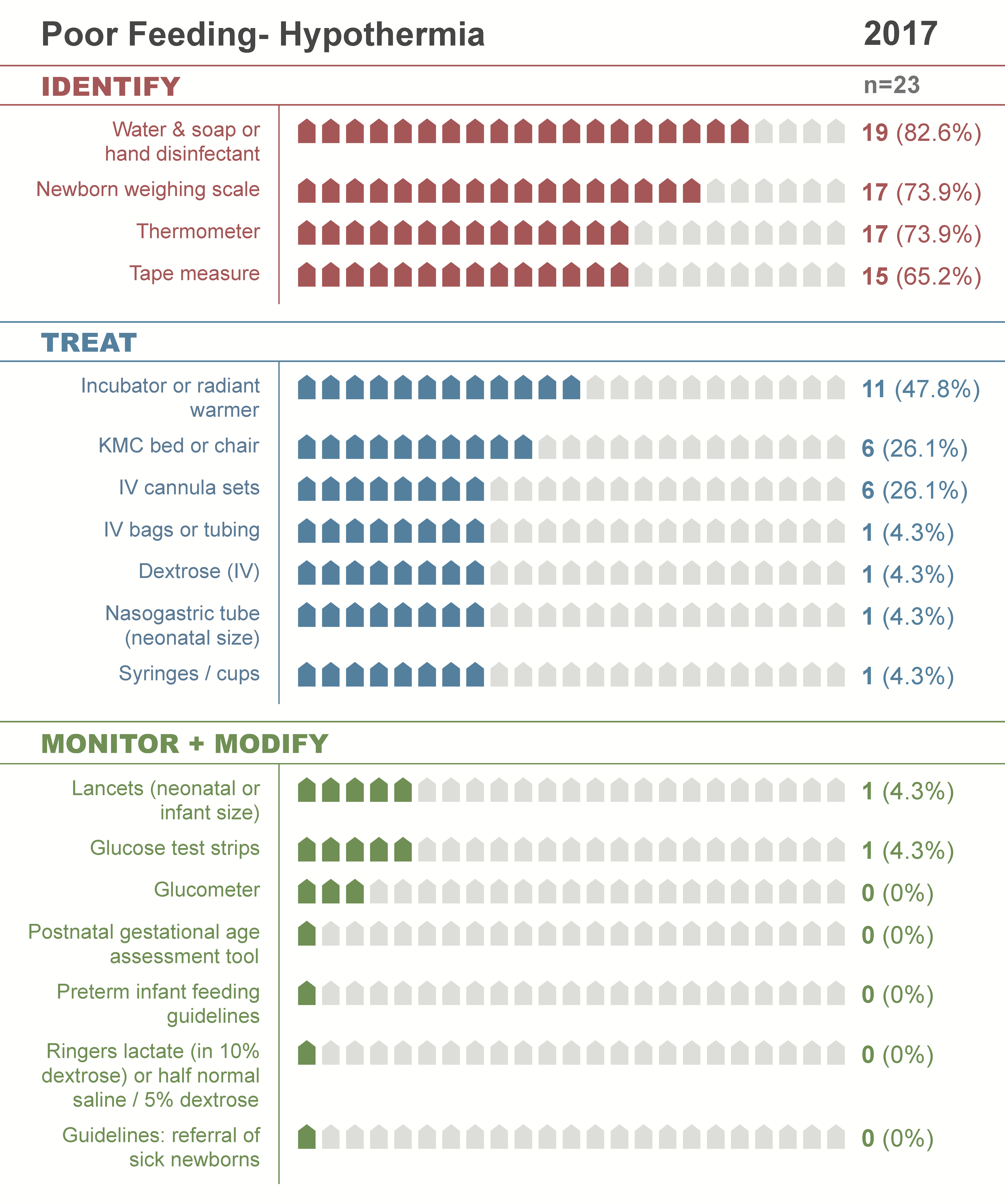

Supplement: S4 Fig — See Table 2 for relevant footnotes. (TIFF) [file pone.0207156.s008.tiff]
